# Serotonergic underpinnings of obsessive‐compulsive disorder: A systematic review and meta‐analysis of neuroimaging findings

**DOI:** 10.1111/pcn.13760

**Published:** 2024-11-07

**Authors:** Martin Pastre, Bob‐Valéry Occéan, Vincent Boudousq, Ismael Conejero, Pascale Fabbro‐Peray, Laurent Collombier, Luc Mallet, Jorge Lopez‐Castroman

**Affiliations:** ^1^ Department of Psychiatry CHU Nimes Nimes France; ^2^ Laboratoire de Biostatistique, Epidémiologie clinique, Santé Publique Innovation et Méthodologie (BESPIM) CHU Nimes Nimes France; ^3^ Département de Médecine Nucléaire et Biophysique Médicale CHU Nimes Nimes France; ^4^ Université Paris‐Est Créteil, DMU IMPACT, Département Médical‐Universitaire de Psychiatrie et d'Addictologie Hôpitaux Universitaires Henri Mondor‐Albert Chenevier, Assistance Publique‐Hôpitaux de Paris Créteil France; ^5^ Institut du Cerveau‐Paris Brain Institute–ICM Sorbonne Université, Inserm, CNRS Paris France; ^6^ Department of Mental Health and Psychiatry, Global Health Institute University of Geneva Geneva Switzerland; ^7^ CIBERSAM, ISCIII Madrid Spain; ^8^ Department of Psychiatry, Radiology, Public Health, Nursing and Medicine University of Santiago de Compostela Santiago de Compostela Spain

**Keywords:** 5‐HT2A binding, molecular imaging, obsessions, serotonin, SERT binding

## Abstract

Obsessive‐compulsive disorder (OCD) is a frequent and disabling condition, with many patients being treatment‐resistant. Improved understanding of its neurobiology is vital for better therapies. Evidence is still conflicting regarding specific serotonergic‐related dysfunctions in OCD. We systematically reviewed the literature to provide a quantitative assessment of the role of serotonin (5‐HT) in patients with untreated OCD through imaging. We searched for neuroimaging studies investigating central 5‐HT tonus in unmedicated patients with OCD, excluding studies comprising treated patients to prevent bias from antidepressant‐induced changes in serotonergic tonus. We also conducted a meta‐analysis using a homogeneous group of positron emission tomography and single photon emission computed tomography articles that compared 5‐HT transporter (SERT) and 5‐HT2A receptor (HT2AR) binding potential in different brain regions of patients with untreated OCD and healthy controls. The systematic review encompassed 18 articles, with 13 included in the subsequent meta‐analysis. Risk of bias was assessed by a revised form of the Newcastle‐Ottawa Scale. We provided standardized mean difference (SMD) values for SERT and 5‐HT2AR binding potential measures across 15 different brain regions. Patients with OCD showed lower SERT binding potential in the brainstem (SMD = −1.13, 95% CI [−1.81 to −0.46]), midbrain (SMD = −0.54, 95% CI [−0.92 to −0.16]), and thalamus/hypothalamus regions (SMD = −0.58, 95% CI [−0.99 to −0.18]) with neglectable to moderate heterogeneity. By combining results from 2 decades of molecular imaging studies, we show that individuals with OCD exhibit lower SERT binding potential in specific brain regions, providing compelling evidence of a 5‐HT system dysfunction. However, the exact mechanisms underlying this phenotype remain elusive. The limitations include heterogeneity across studies in populations, imaging techniques, and radiotracer usage.

Obsessive‐compulsive disorder (OCD) is a prevalent and disabling condition, affecting approximately 1.2% of the general population in the United States and between 1.1% and 1.8% worldwide.^1^ The robust therapeutic response to selective serotonin reuptake inhibitors (SSRIs) is the clearest finding in the OCD treatment literature, despite the fact that around 40% to 60% of patients do not respond to the initial SSRI treatment.^2^ Therefore, characterizing the dynamics of endogenous serotonin (5‐HT) remains a crucial goal. Yet, such characterization is limited because brain 5‐HT cannot be directly assayed in humans.

The 5‐HT hypothesis, based on the relative efficacy of clomipramine and SSRIs in treating OCD, finds some support in other research. An early study by Thoren et al. in 1980 revealed an association between improved OCD symptoms with clomipramine treatment and a decrease in a 5‐HT metabolite (5‐HIAA) in the cerebrospinal fluid.^3^ The cortico‐striato‐thalamo‐cortical (CSTC) circuitry, which is consistently found to be dysregulated in OCD,^4^, ^5^, ^6^, ^7^ contains serotonergic axons projecting from the raphe nuclei.^8^ Furthermore, certain polymorphisms in 5‐HT system genes, notably the 5‐HTTLPR of the 5‐HT transporter (SERT) gene and the rs6311 of the 5‐HT2A receptor (5‐HT2AR) gene, have been associated with OCD. However, the overall results from studies investigating these associations have been inconclusive.^9^

Similarly, pharmacological challenge studies using sumatriptan (a 5‐HT1D agonist) and mCPP (an agonist of the 5‐HT receptor family) have produced inconclusive results in inducing OCD symptoms in patients with OCD,^10^, ^11^, ^12^, ^13^ and tryptophan depletion strategies failed to exacerbate these symptoms.^14^ Non‐SSRI serotoninergic agents such as buspirone (a 5‐HT1A partial agonist) and ondansetron (a 5‐HT3 antagonist) have been investigated as potential OCD treatments, but their effectiveness remains uncertain.^5^, ^15^

While positron emission tomography (PET) and single‐photon emission computed tomography (SPECT) have made significant strides, the challenge of reconciling conflicting evidence on serotonergic‐related dysfunction in OCD persists.^4^, ^5^, ^8^, ^15^, ^16^ Moreover, a complex interaction among neurotransmission systems, including glutamatergic and dopaminergic systems, may be at play in OCD.^4^, ^15^ Recent meta‐analyses in molecular imaging have explored dopamine changes in OCD and anxiety disorders with inconclusive results.^17^

Several recent reviews and meta‐analyses have compiled neuroimaging findings in OCD from magnetic resonance spectroscopy,^18^, ^19^ functional magnetic resonance imaging (fMRI),^7^, ^20^ PET and SPECT imaging,^21^ and diffusion tensor imaging.^22^ However, there are no recent systematic reviews specifically assessing serotonergic mechanisms through imaging. A parallel exploration, a meta‐analysis on brain molecular imagery of SERT, has been performed in the context of major depressive disorder.^23^ This paper aims to systematically review the literature and provide a high‐quality quantitative assessment of the role of 5‐HT in OCD through imaging, shedding light on the serotonergic system's involvement in this disorder.

## Methods

### Selection of studies

We selected all studies according to the following eligibility criteria: (i) original studies published until August 2023 in English, French, or Spanish language; (ii) the study samples comprised adult patients with an OCD according to the DSM‐III, IV, or 5 criteria; and (iii) the studies used neuroimaging data investigating central 5‐HT tonus in unmedicated patients with OCD. We have chosen to utilize the term “5‐HT tonus” through the paper as the most appropriate descriptor for the overall state of various 5‐HT receptors or transporters, which emerge from distinct tracers and are subject to competition for 5‐HT binding. This term does not directly indicate the levels of serotonin itself or the activity of serotonin neurons. 5‐HT tonus could be evaluated through PET and SPECT analyses of SERT binding potential, 5‐HT1A/2A/3/1B/1D receptors binding potential and regional synthesis of 5‐HT, or other imaging techniques investigating the connectivity or morphology of structures that have a high‐density of serotonergic neurons, e.g. the raphe nuclei of the midbrain. We excluded imagery studies comprising treated patients to avoid the bias linked to the modification of the 5‐HT tonus under antidepressant treatment or 5‐HT2AR agonists, as well as magnetic resonance spectroscopy studies that have been systematically reviewed recently.^19^ Reviews and meta‐analyses were also excluded.

We followed the PRISMA (Preferred Reporting Items for Systematic Reviews and Meta‐Analyses) 2020 checklist for systematic reviews and published the protocol on the PROSPERO registry for systematic reviews (CRD42022373373).

### Data sources and search strategy

To identify potential papers, we searched two databases: PubMed and Web of Science until January 2024 (see Supplementary Material for search equation terms). The reference lists of the selected studies and recent narrative reviews on the topic were also examined to search for additional records. We searched for unpublished results from open gray databases (doctoral theses, research abstracts, and presentations). Two reviewers working independently (M.P. and J.L.C.) screened the title and abstract of each potential paper using Zotero software and the Rayyan platform for the management of records. The full text of eligible studies was then reviewed independently by the same two reviewers to assess all inclusion and exclusion criteria. In the event of disagreement, a third author was committed to rule a decision (P.F.P.).

### Data extraction and quality assessment

Using a data chart, we extracted the following data from each selected paper: (i) first author's name, journal, and date of publication; (ii) number of participants, gender distribution, drug status, mean Yale‐Brown Obsessive‐Compulsive Scale (Y‐BOCS score, comparison group, and OCD subtype if stated; (iii) imagery method, principal outcomes, and region of reference for the analysis; and (iv) the main results with estimates, SDs, and *P*‐values.

We extracted the radioligand binding potential values for each region of interest (ROI) in each study included in the meta‐analysis (data available upon request). Different outcomes reflecting the same physical variable are often used in studies investigating radioligand binding in the brain. They all rely on a three‐compartment model (see Supplementary Material). If two different types of measurements were provided by the authors (e.g. V3″ and BP)^24^ we chose the one most frequently employed in the literature to make comparisons easier. One study provided distinct SERT binding potential values for early‐onset versus late‐onset OCD.^25^ Therefore, we used the Cochrane formula for combining groups.^26^

For the meta‐analysis, all articles were meticulously reviewed by trained nuclear physicists (V.B. and L.C.) to ensure comparability. To assess the quality of each study individually, two different psychiatrists (M.P. and J.L.C.) conducted an independent evaluation using a modified version (Table 2) of the Newcastle‐Ottawa Scale for case‐control studies from Wells et al.^42^ Specifically, we excluded item 4 from the selection subpart as it was not applicable to the imaging studies, along with items 2 and 3 from the outcome/exposure subpart, which pertain to follow‐up measures.

### Meta‐analysis

To ensure meaningful and physiologically relevant results, we conducted separate meta‐analyses for different brain regions, specifically focusing on brain regions that appeared in two or more studies. The complete strategy used for the grouping of brain ROIs is presented in the Supplementary Methods.

To measure the effect sizes, we used standardized mean differences (SMDs). Both standard and random‐effects models were specified to account for between‐study variance. To pool the results, we employed inverse variance weighting, which maximizes the accuracy of the common effect estimate while minimizing variance. To assess heterogeneity between studies, we utilized *I* ^2^ statistics, which estimate the percentage of total variation across studies attributable to heterogeneity rather than chance. We considered *I* ^2^ values of 25%, 50%, and 75% as indicators of low, moderate, and substantial heterogeneity, respectively. To ensure robustness of the findings, we performed a sensitivity analysis, excluding studies with a high risk of bias. We furthermore performed leave‐one‐out analyses to assess the influence of each individual study on the overall results (data are available upon request). The interpretation of SMD effect sizes was as follows: small (0.2 to <0.3), moderate (0.3 to <0.8), and large (>0.8). For statistical analysis, we utilized the General Package for Meta‐Analysis “meta” version 6.1‐0 in R software version 4.0.0.

## Results

**Fig. 1:**
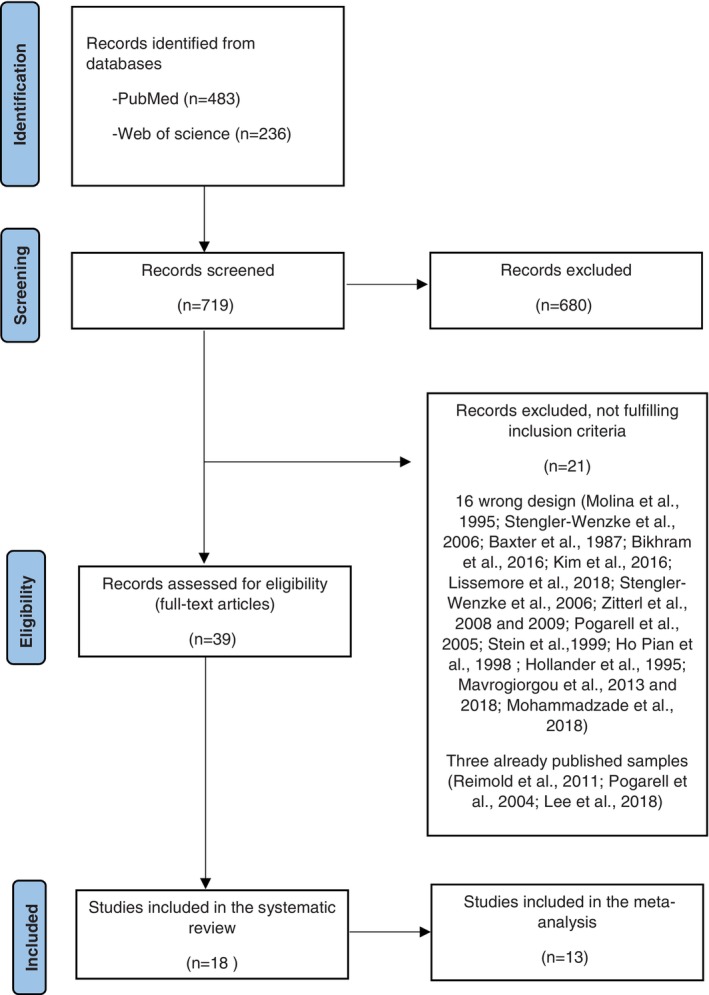
Flowchart of the selected studies.

The search yielded a total of 719 potential papers, with 483 retrieved from PubMed and 236 from the Web of Science. After a thorough review of titles and abstracts, 680 papers were excluded because of their lack of relevance to the topic or duplication. Among the remaining 39 papers that underwent a full assessment for eligibility, 21 were excluded as they did not meet the criteria. Notably, three papers were excluded because they presented results that were partly already described in other included publications.^43^, ^44^, ^45^ We also excluded six articles that involved treated individuals in a “pre‐post treatment” design, examining the effect of antidepressant medication on SERT binding potential in patients with OCD,^46^, ^47^, ^48^ or employed a within‐subject design without a control group.^49^ Figure 1 presents a flow diagram with the reasons for exclusion. For the systematic review, a total of 18 articles were included (Table 1).

**Table 1:** Study characteristics and main findings

Publication	Inclusion status	Study design and sample	Imaging methods (outcome)	Measure (binding)	Main results
Reimold et al.^32^	Systematic review + meta‐analysis	Case‐control; 9 OCD and 19 age‐, sex‐, genotype‐, and smoking status–matched HCs	[^11^C]DASB PET (BP_ND_)	SERT	Lower SERT binding potential in the midbrain (2.44 ± 0.33 for OCD vs 2.87 ± 0.58 for HCs) and thalamus (1.14 ± 0.24 for OCD vs 1.35 ± 0.18) in OCD vs HCs Positive association between SERT binding potential values and Y‐BOCS scores (thalamus binding potential and age explained 83% of Y‐BOCS variance)
Matsumoto et al.^29^	Systematic review + meta‐analysis	Case‐control; 10 OCD and 18 age‐ and sex‐matched HCs	[^11^C]DASB PET (BP_ND_)	SERT	Lower SERT binding potential in the OFC (0.13 ± 0.05 for HCs vs 0.08 ± 0.02 for OCD; *P* = 0.0005), the temporal cortex (0.43 ± 0.12 vs 0.34 ± 0.03; *P* = 0.007) and the insular cortex (0.43 ± 0.11 vs 0.30 ± 0.04; *P* = 0.0008)
Hesse et al.^25^	Systematic review + meta‐analysis	Case‐control; 19 (13 late‐onset/and 6 early‐onset) OCD and 21 HCs	[^11^C]DASB PET (BP_ND_)	SERT	Lower binding potential in LO‐OCD vs HCs in the raphe, midbrain, hypothalamus, thalamus, striatum, hippocampus, amygdala, medial prefrontal cortex, and occipital cortex Lower binding potential in LO‐OCD vs EO‐OCD in the midbrain, thalamus, striatum, hippocampus, and occipital cortex
Pogarell et al.^31^	Systematic review + meta‐analysis	Case‐control; 9 OCD and 10 HC	[^123^I]‐β‐CIT SPECT (BP_ND_)	SERT	Higher SERT binding potential values in the midbrain‐pons region for OCD (2.26 ± 0.37 vs 1.81 ± 0.23 for HCs, *P* = 0.003)
Stengler‐Wenzke et al.^34^	Systematic review + meta‐analysis	Case‐control; 10 OCD and 7 age‐matched HCs	[^123^I]‐β‐CIT SPECT (BP_ND_)	SERT	Lower SERT binding potential in the midbrain (3.51 ± 0.45 vs. 4.89 ± 1.23; *P* < 0.005) and the brainstem (2.38 ± 0.76 vs 3.53 ± 1.01; *P* < 0.05)
Van der Wee et al.^35^	Systematic review + meta‐analysis	Case‐control; 15 OCD and 15 age‐ and sex‐matched HCs	[^123^I]‐β‐CIT SPECT (BP_ND_)	SERT	No difference in SERT binding potential between OCD and HCs
Hesse et al.^28^	Systematic review + meta‐analysis	Case‐control; 15 OCD and 10 HCs	[^123^I]‐β‐CIT SPECT (BP_ND_)	SERT	Lower SERT binding potential in the thalamus/hypothalamus (*P* = 0.026), midbrain (*P* = 0.008), and brainstem regions (*P* = 0.014) of OCD Negative association between SERT binding potential in the hypothalamus/thalamus and Y‐BOCS scores (*r* = −0.79, *P* = 0.001)
Hasselbalch et al.^50^	Systematic review + meta‐analysis	Case‐control; 9 OCD and 9 HCs	[^123^I]‐β‐CIT SPECT (BP_ND_)	SERT	Lower SERT binding potential in midbrain‐pons of OCD (0.97 ± 0.07 vs 0.84 ± 0.12; *P* = 0.011)
Zitterl et al.^36^	Systematic review + meta‐analysis	Case‐control; 24 OCD (checking compulsions) and 24 age‐ and sex‐matched HCs	[^123^I]‐β‐CIT SPECT (BP_ND_)	SERT	Lower SERT binding potential values in the thalamus/hypothalamus of OCD (1.38 ± 0.19 vs 1.69 ± 0.21; *P* < 0.001) Negative correlation between SERT binding potential in the thalamus/hypothalamus region and Y‐BOCS score (*r* = −0.80, *P* < 0.001) Impact of severity of illness and duration of illness on SERT binding potential values (*β* = −0.03 ± 0.005, *P* < 0.001; β0.006 ± 0.002, *P* < 0.05, respectively)
Müller‐Vahl et al.^40^	Systematic review only	Case‐control; 8 TS + OCD, 5 pure OCD, and 10 HCs	[^123^I]‐ADAM SPECT (BP_ND_)	SERT	Higher SERT BP_ND_ in the caudate nucleus (*P* = 0.0284), hypothalamus (*P* = 0.0227), and midbrain (*P* = 0.0191) of TS + OCD vs HCs Higher BP_ND_ in the caudate nucleus (*P* = 0.0216), midbrain (*P* = 0.0479), and thalamus (*P* = 0.0491) for TS + OCD vs TS‐OCD
Simpson et al.^24^	Systematic review + meta‐analysis	Case‐control; 11 OCD and 11 age‐, sex‐, and ethnicity‐matched HC	[^11^C](+)McN‐5652 PET (BP_P_)	SERT	No difference in SERT binding potential between OCD and HCs
Wong et al.^38^	Systematic review only	Case‐control; 9 TS + OCD, 2 TS‐OCD, and 9 HCs	[^11^C](+)McN‐5652 for SERT	SERT; 5‐HT2A	Lower SERT binding potential in the midbrain (*P* < 0.05) of TS + OCD compared with HCs No significant difference between groups (TS + OCD vs TS‐OCD vs HCs) in regard to 5‐HT2A binding potential
			[^11^C]MDL for 5‐HT2A PET (BP_ND_)		
Perani et al.^30^	Systematic review + meta‐analysis	Case‐control; 9 OCD and 15 age‐matched HCs	[^11^C]MDL PET (BP_ND_)	5‐HT2A	Lower binding potential across multiple cortical regions (frontal and cingulate cortices) Inverse association between Y‐BOCS score and 5‐HT2A binding potential in frontal and temporal cortices of patients
Simpson et al.^33^	Systematic review + meta‐analysis	Case‐control; 19 OCD and 19 age‐, sex‐, ethnicity‐, and smoking status–matched HCs	[^11^C]MDL PET (BP_ND_)	5‐HT2A	No difference in 5‐HT2A binding potential across groups Inverse relationship between 5‐HT2A binding potential in OFC and age at onset in patients with OCD (Pearson *ρ* = −0.68, *P* = 0.002)
Pittenger et al.^39^	Systematic review only	Case‐control; 12 OCD and 12 age‐, sex‐, and BMI‐matched HCs	[^11^C]p943 PET (BP_ND_)	5‐HT1B	No difference in 5‐HT2A binding potential between OCD and HCs
Adams et al.^27^	Systematic review + meta‐analysis	Case‐control; 15 OCD and 15 age‐ and sex‐matched HCs	[^18^F]altanserin PET (BP_P_)	5‐HT2A	Higher 5‐HT2A binding potential in the caudate nucleus of OCD (0.24 ± 0.14 vs 0.15 ± 0.13, *P* < 0.05)
Berney et al.^37^	Systematic review only	Case‐control; 21 OCD and 21 age‐ and sex‐matched HCs	Alpha[^11^C]methyl‐L‐tryptophan PET (trapping constant K*)	5‐HT synthesis	Higher K* in the right hippocampus [F (1, 40) = 14.75 (*P* < 0.001)] and inferior temporal gyrus [F (1, 40) = 7.97 (*P* < 0.007)] of OCD In the subgroup of men only, higher K* values in the caudate nuclei of OCD (F (2, 39) = 9.06, *P* < 0.01) Positive association between K* in the temporal gyri and Y‐BOCS in patients
Kim et al.^41^	Systematic review only	Case‐control; 102 OCD and 101 age‐ and sex‐matched HCs	Resting‐state fMRI (FC with RN as seed ROI)	NA	Higher FC between RN and the temporal cortex, amygdala, hippocampus, putamen, caudate, thalamus, and brainstem of OCD Lower FC in the left occipital pole of OCD Greater FC linking RN and medio‐superior temporal gyri in nonresponders as compared with responders (*P* < 0.05) A smaller FC linking RN and medio‐superior temporal gyri was a predictor of clinical improvement

5‐HT2AR, 5‐HT2A receptor; BMI, body mass index; BP_ND_, binding potential referred to the nondisplaceable compartment in brain; BP_P_, binding potential referred to plasma concentration; EO, early‐onset; FC, functional connectivity; fMRI, functional magnetic resonance imaging; HC, healthy control; LO, late‐onset; NA, not available; OCD, obsessive‐compulsive disorder; OFC, orbitofrontal cortex; PET, positron emission tomography; RN, raphe nuclei; ROI, region of interest; SERT, serotonin transporter; SPECT, single‐photon emission computed tomography; TS, transcranial sonography; Y‐BOCS, Yale‐Brown Obsessive‐Compulsive Scale.

Five records were systematically reviewed but excluded from the meta‐analysis. Two of them were molecular imaging studies but focused on patients with Tourette syndrome.^38^, ^40^ Despite the relevance of OCD comorbidity in their samples, the authors did not provide usable values of 5‐HT binding for patients with OCD. Efforts to contact the authors by email did not yield a response. The meta‐analysis ultimately comprised 13 articles, with 10 studies investigating SERT binding potential across different ROIs in medication‐free patients with OCD versus healthy controls (HCs), and three studies investigating 5‐HT2AR binding in the same populations.

Across studies, the mean ages of the patients ranged from 25.3 years^41^ to 44 years.^28^ The mean Y‐BOCS scores varied from 17^40^ to 30,^27^ representing a range from moderate to severe symptoms of OCD. Molecular imaging studies are categorized based on the radiotracer used and the date of publication in Table 1. We only present statistically significant results. A detailed description of studies investigating SERT and 5‐HT2A binding potential is provided in the Supplementary Material, as well as a description of serotonin synthesis and fMRI studies. A quality assessment of all of the studies included in the systematic review is presented in Table 2. The quality of the studies is overall good, but two studies (Hesse et al.^28^ and Stengler‐Wenzke et al.^34^) appeared to be less qualitative in regard to their scores on the Newcastle‐Ottawa Scale (i.e. scoring two on six possible stars). We therefore performed sensitivity analyses by excluding these two studies to assess the robustness of our findings, which retrieved similar results compared to the less restrictive analysis (SMD = −0.54 [−1.05 to −0.02] for SERT binding in the thalamus/hypothalamus subregion after excluding the latter two studies, see Fig. S5).

**Table 2:** Quality assessment of studies included in the systematic review

Quality assessment: Newcastle‐Ottawa Scale							
	Selection			Comparability	Outcome	Total	Journal
Article	**1**	**2**	**3**				
Adams et al.^27^	X	0	X	XX	X	5	*International Journal of Neuropsychopharmacology*
Hasselbach et al.^50^	X	0	X	XX	0	4	*Acta Psychiatrica Scandinavica*
Hesse et al.^28^	X	0	0	X	0	2	*Psychiatry Research: Neuroimaging*
Hesse et al.^25^	X	0	0	XX	0	3	*International Journal of Neuropsychopharmacology*
Matsumoto et al.^29^	X	0	X	XX	0	4	*NeuroImage*
Perani et al.^30^	X	X	X	X	0	4	*NeuroImage*
Pogarell et al.^31^	X	X	0	XX	X	5	*Biological Psychiatry*
Reimold et al.^32^	X	0	0	XX	0	3	*Journal of Neural Transmission*
Simpson et al.^24^	X	0	X	XX	X	5	*Biological Psychiatry*
Simpson et al.^33^	X	0	X	XX	X	5	*Biological Psychiatry*
Stengler‐Wenzke et al.^34^	X	0	0	X	0	2	*European Archives of Psychiatry and Clinical Neurosciences*
Van der Wee et al.^35^	X	0	X	XX	X	5	*American Journal of Psychiatry*
Zitterl et al.^36^	X	X	0	XX	X	5	*Neuropsychopharmacology*
Berney et al.^37^	X	0	X	XX	X	5	*Archives of General Psychiatry*
Wong et al.^38^	X	0	X	X	0	3	*Neuropsychopharmacology*
Pittenger et al.^39^	X	0	X	XX	0	4	*Journal of Affective Disorders*
Müller‐Vahl et al.^40^	X	0	0	XX	0	3	*Scientific Reports*
Kim et al.^41^	X	0	X	XX	0	4	*Neuropsychopharmacology*

*Selection*: 1. One point is given if the case definition is adequate (i.e. a validated diagnostic scale is used to identify cases). 2. One point is given if all eligible cases present the outcome of interest over a defined period of time, all cases in a defined catchment area, all cases in a defined hospital or clinic, group of hospitals, health maintenance organization (e.g. consecutive recruitment of new patients with obsessive‐compulsive disorder), or an appropriate sample of those cases (e.g. random sample). 3. One point is given if the control subjects issue from the community. *Comparability*: One point is given if controls are matched by age or analyses are adjusted by age; two points if an adjustment is made by any additional factor. Cases and controls must be matched in the design and/or confounders must be adjusted for in the analysis. *Outcome*: One point if the investigators are blind to the results of imagery assessments (positron emission tomography, single‐photon emission computed tomography, or magnetic resonance imaging). *If quality items are not specified in the studies*, *they are scored 0*.

The funnel plots did not show asymmetrical patterns and did not convey an eventual publication bias (Figs S1,S2). The linear regression test of funnel plot asymmetry (Egger test,^51^) was not applicable because of the low number of studies included in our meta‐analysis. The Baujat plot did not indicate outliers that should be excluded from the analyses (see Fig. S3).

### SERT studies

A total of 12 studies investigated the SERT binding potential *via* molecular imaging in patients with OCD.

#### Meta‐analytic results (SERT binding)

**Fig. 2:**
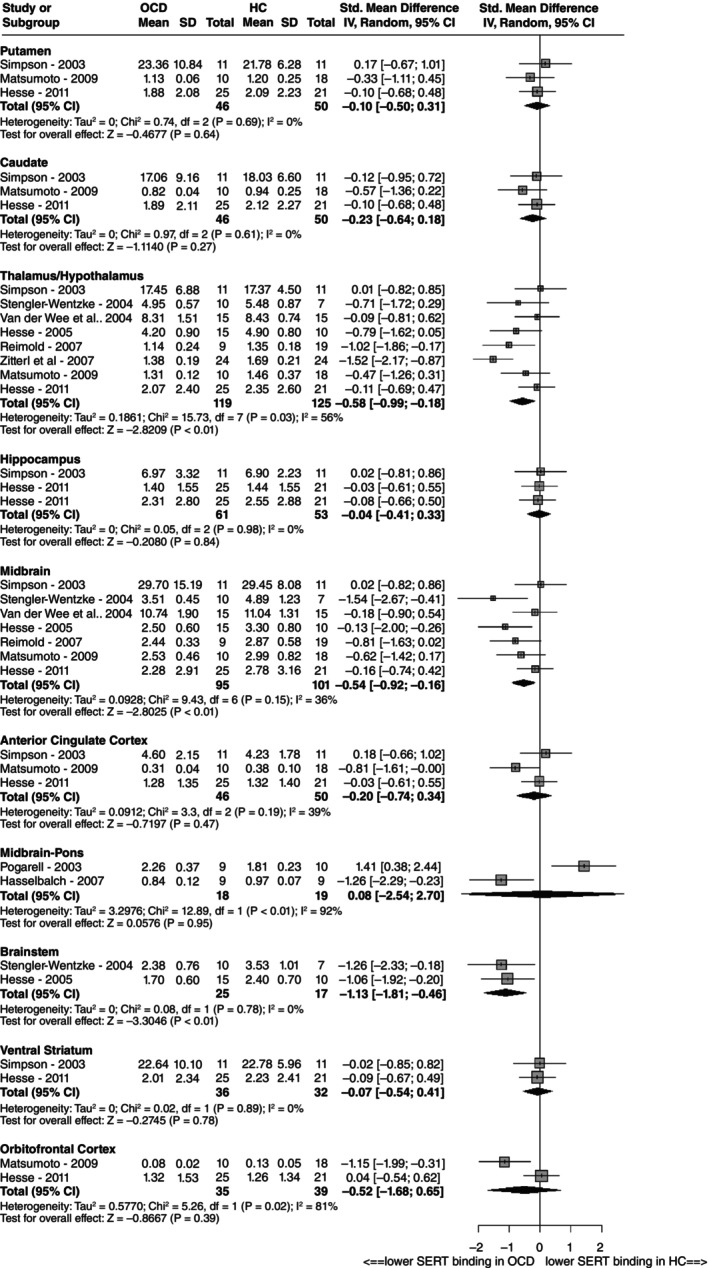
Meta‐analytic results of serotonin transporter binding: Forest plots for each region of interest subgroup. CI, confidence interval; HC, healthy control; OCD, obsessive‐compulsive disorder.

The SERT binding potential was lower in the brainstem region, with a large effect size (SMD = −1.13 [−1.81 to −0.46]) and neglectable heterogeneity (*I* ^2^ = 0%), based on two studies (comprising 25 individuals with OCD and 17 controls) (see Fig. 2). Concerning the midbrain region, we found a moderate effect size towards lower SERT binding potential, with an SMD of −0.54 (−0.92 to −0.16) based on a sample from seven studies (95 patients with OCD and 101 HCs), showing low to moderate heterogeneity (*I* ^2^ = 36%). The SERT binding potential was also lower in the thalamus/hypothalamus region, with moderate effect size (SMD = −0.58 [−0.99 to −0.18]) and moderate heterogeneity (*I* ^2^ = 56%) based on a sample from eight studies (119 patients with OCD and 225 controls). We conducted a sensitivity analysis including only studies focusing solely on the thalamus (*n* = 4), which retrieved a similar effect size despite not reaching significance (SMD = −0.35 [−0.78 to 0.07]), presumably because of the lack of an adequate sample size (see Fig. S4). In addition, we observed a lower heterogeneity (*I* ^2^ = 23%), likely attributable to consistent study designs and measurement techniques. Concerning midbrain‐pons regions, there was very high heterogeneity (*I* ^2^ = 92%), with two studies showing opposite effects. Although they were not statistically significant, we also observed lower binding potential values for patients with OCD versus HCs in the orbitofrontal cortex (OFC; SMD = −0.52 [−1.68 to 0.65]; *I* ^2^ = 81%), the caudate (SMD = −0.23 [−0.64 to 0.18]; *I* ^2^ = 0%), and the anterior cingulate cortex (ACC; SMD = −0.20 [−0.74 to 0.34]; *I* ^2^ = 39%).

### 5‐HTR studies

Five studies investigated 5‐HTR binding potential using PET imaging. Four of them focused on 5‐HT2AR^27^, ^30^, ^33^, ^38^ and one on 5‐HT1D binding.^39^

#### Meta‐analytic results (5‐HT2AR binding)

**Fig. 3:**
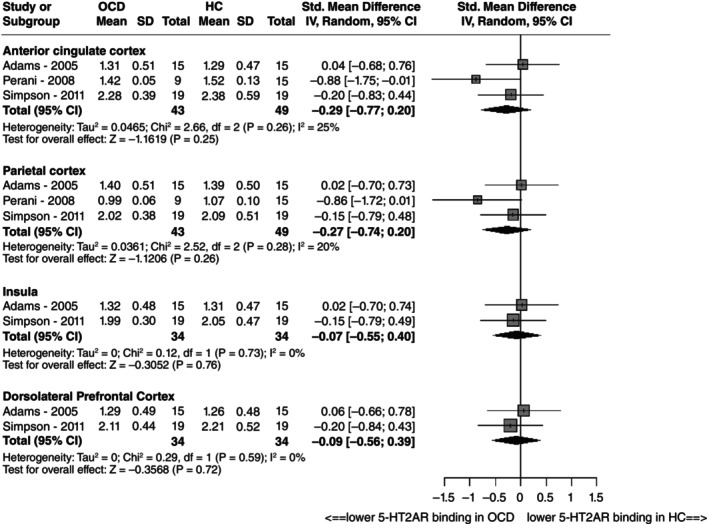
Meta‐analytic findings of 5‐HT2A receptor binding. Forest plots for each region of interest subgroup. CI, confidence interval; HC, healthy control; OCD, obsessive‐compulsive disorder.

Heterogeneity among subregions was minimal (*I* ^2^ ranged from 0 to 25%) (see Fig. 3). Two ROIs exhibited small effect sizes: the ACC (SMD = **−**0.29 [−0.77 to 0.20]) and the parietal cortex (SMD = **−**0.27 [−0.74 to 0.20]), demonstrating lower 5‐HT2AR binding potential values in the OCD group. However, these outcomes did not achieve statistical significance.

## Discussion

Our meta‐analytic findings indicate that unmedicated individuals with OCD exhibit lower SERT binding in the brainstem and the midbrain. Lower SERT binding in these regions provides compelling evidence for 5‐HT system dysfunction. This is especially significant since the brainstem houses the raphe nuclei, which contain the neuronal cell bodies of serotonergic neurons.^52^ The reduction in SERT binding in the midbrain may indicate either a primary decrease in the number of cell bodies within the raphe nuclei,^50^ or it could result from a more intricate regulatory mechanism involving somatodendritic transporters.^53^ However, two studies did not find variations in SERT binding in the pons and raphe nuclei regions, respectively.^28^, ^35^ We could not include these findings in our meta‐analysis since these regions were not investigated in other studies, and we chose not to regroup them with midbrain and brainstem regions for the reasons explained earlier.

Patients with OCD also displayed lower SERT binding in the thalamus and hypothalamus regions. The moderate heterogeneity identified in our meta‐analysis regarding this specific brain region (*I* ^2^ = 36%) may be attributed to differences in outcome measurement methods among the studies. Indeed, apart from studies focusing solely on the thalamus,^24^, ^29^, ^32^, ^35^ other studies chose to provide pooled measures of thalamic and hypothalamic regions^36^ or to delineate a single ROI encompassing thalamic and hypothalamic regions.^25^, ^34^ One team chose to measure two different binding values for the thalamic and hypothalamic regions (see the Section Methods).^25^ Despite being anatomically close and part of the limbic system, these two regions have different functions, which calls into question the validity of grouping them together.^25^

In two studies investigating SERT binding before and after treatment, there was a correlation between symptomatic improvement and the reduction of SERT binding under antidepressant pharmacotherapy in thalamus/hypothalamus.^46^, ^47^ As mentioned by the authors, the observed baseline reduction in SERT binding within the thalamus/hypothalamus may be interpreted as a compensatory mechanism triggered by decreased 5‐HT levels. This mechanism would raise intrasynaptic 5‐HT concentrations by reducing the rate of reuptake. Likewise, the subsequent reduction in SERT binding facilitated by antidepressants would further enhance 5‐HT levels in the synaptic cleft, resulting in more signal transduction and thus promoting symptom remission via enhanced neuromodulation tonus. In our view, these dynamic findings regarding SERT binding should be considered alongside the results of Berney et al.,^37^ who reported heightened 5‐HT synthesis in the hippocampal and inferior temporal regions of patients with OCD.^29^ In a subsequent study, Lissemore et al.^54^ demonstrated that treatment with sertraline or cognitive behavioral therapy (CBT) resulted in increased α‐[^11^C]MTrp trapping in responders, in the same regions that exhibited high 5‐HT synthesis at baseline.^54^ Based on these results, the authors proposed a “braking system model of OCD,” suggesting that the elevated brain regional 5‐HT synthesis observed in patients with OCD might represent an unsuccessful attempt by the organism to regain control over obsessions and compulsions. According to this hypothesis, drug therapies could potentially enhance the impaired “braking system” in OCD.^8^, ^54^

We also found lower SERT binding in the OFC of patients with OCD. Although statistically nonsignificant, this result showed a moderate size effect (SMD = −0.52). The heterogeneity was high for this ROI (*I* ^2^ = 81%), presumably because of variations in patient populations (the patients in Hesse et al.^25^ only had checking symptoms, unlike those in Matsumoto et al., who mostly received CBT when the data were collected)^25^, ^29^ or study design (SPECT versus PET with different radioligands). Accordingly, and despite statistical nonsignificance, patients with OCD showed lower SERT binding in the caudate and the ACC. The small number of patients in the analysis might be the reason why these ROIs show moderate SMD effect sizes but no statistical significance. Future studies with more patients are needed to conclusively evaluate SERT binding in these regions.

The crucial role of OFC, ACC, caudate, and thalamus regions in the pathophysiology of OCD, according to the CSTC pathway model, has been extensively established. Early studies showed that these regions exhibit increased glucose metabolic rate in PET and SPECT studies (see Nakao et al.^7^ for a review)^7^, ^55^ and overactivation in fMRI studies.^56^ In addition, structures of orbitofrontal and ACC loops display increased activity during OCD symptom provocation.^57^ Interestingly, the overactivation in OFC, caudate, and thalamus is reversed by SSRI and CBT treatments.^21^ These findings confirm the role of the aforementioned regions in OCD pathophysiology and support the hypothesis of a hyperactive orbitofrontal‐striatal pathway in OCD, which SSRIs successfully mitigate. Our observation of reduced SERT binding in these regions, considering their dense serotonergic innervation from the midbrain's raphe nuclei, leads us to propose a potential connection between the “braking system model” and the classical CSTC pathways model. The 5‐HT system might, in physiological conditions, adjust the balance between direct and indirect CSTC pathways. Neuromodulation is presumably weakened in OCD, and the decrease in SERT binding observed at baseline may reflect an unsuccessful attempt of the organism to increase 5‐HT tonus across CSTC structures.

Regarding 5‐HT2AR binding, two ROIs displayed minor effect sizes. In the ACC and the parietal cortex, 5‐HT2AR binding values appeared lower in the OCD group; however, these outcomes also lacked statistical significance. Perani et al.^30^ speculated that the reduced 5‐HT2AR binding in the cortical regions of patients with OCD is a signal of prolonged receptor downregulation caused by insufficient 5‐HT release. They additionally conjectured that this discovery might indicate an inherent scarcity in 5‐HT2ARs, potentially stemming from genetic variations. Moreover, Adams et al.^27^ identified heightened 5‐HT2AR binding in the caudate nucleus—a unique finding exclusive to their study and thus not integrated into our meta‐analysis. The caudate nucleus has previously been associated with OCD's underlying processes. Notably, their study is the only one we are aware of that demonstrates elevated 5‐HT2A binding in the brains of patients with OCD versus controls. The authors propose that this outcome might reflect a secondary, adaptive mechanism—a rise in receptor density due to diminished 5‐HT levels within the CSTC system. However, such a result must be taken cautiously, given the very low density of 5‐HT2A in that brain region.

In addition to the meta‐analytic results, our systematic review comprised five more studies. Two of them were molecular imaging studies investigating SERT and 5‐HT2AR binding in a population of patients with Tourette syndrome and comorbid OCD that showed contradictory findings.^38^, ^40^ In addition to Berney et al.^37^ described above,^29^ Pittenger et al. also used molecular imaging but could not be included in our meta‐analysis, because it is the only study to examine 5‐HT1B binding.^39^ Although no significant difference in binding was observed between individuals with OCD and HCs, the authors highlight that the inverse pattern of association between prepulse inhibition and [^11^C]p943 binding across groups could indicate a rearrangement or reshuffling of the serotonergic system in the context of OCD.^15^ Finally, one study reported elevated connectivity between the raphe nuclei and various brain regions along the CSTC pathway in patients with OCD.^41^ The increase in functional connectivity was found to be positively correlated with the severity of the illness, suggesting a significant involvement of serotonergic neurons from the raphe nuclei in the pathophysiology of OCD. These results are consistent with previous literature showing that CSTC structures are hyperconnected within each other and that this connectivity decreases under antidepressant medication.^58^

It is important to note that the differences in binding potential in studies related to serotonergic systems can be interpreted in various ways. Binding potential is often used to reflect the availability of a specific target, assumed to vary directly with 5‐HT receptors or SERT density in each brain structure. However, the binding potential is also influenced by factors such as the affinity of the radioligand used, which can vary between studies, and the presence of the endogenous ligand. As already pointed out by Kambeitz et al.,^58^ altered SERT binding could derive from altered levels of 5‐HT *via* endogenous displacement, when using a tracer competing with 5‐HT for binding (e.g. [^123^I]‐β‐CIT).^23^ Still, studies included in our work, employing radiotracers assumed not to be displaced by endogenous 5‐HT (e.g. [^11^C]DASB),^59^ showed a similar pattern of reduction.^28^, ^29^, ^32^

This study has some limitations. First, heterogeneity across studies in the meta‐analysis could be explained by methodological discrepancies. These studies consisted of PET and SPECT imaging measures, with eight different radiotracers, and differed in ROIs and reference regions chosen for the analysis. The various radiotracers used have shown inequal affinities for the SERT.^60^ Second, although [^123^I]‐β‐CIT is recognized to primarily bind to SERT in hypothalamic/thalamic and midbrain regions,^47^, ^61^ there might still be residual binding to the dopamine transporter due to the affinity profile of this radioligand, potentially introducing bias, particularly in a disorder where dopamine involvement is hypothesized. Moreover, there have been advances in imaging technology since most of the included studies were performed, so that spatial resolution might have been a possible confounding factor in the analyses. There have also been advances in radiochemistry leading to more sensitive tracers for the targets examined here, such as [^11^C]AFM for SERT, warranting further studies in the field using these techniques.^62^ Another factor contributing to heterogeneity is the variation in study populations: Zitterl et al.^36^ was the only study to select a clinically homogeneous sample of patients with checking compulsions.^36^ Accordingly, the distinction between early‐onset and late‐onset OCD was made by only two teams despite its clinical relevance,^63^ and both found that late onset of the illness was associated with lower SERT binding values.^25^, ^31^ Hasselbach et al. and Zitterl et al.^36^ statistically took into account age at onset but did not retrieve any association with SERT binding values.^36^, ^50^ Given the high heterogeneity of OCD, examining clinical subtypes will be crucial in future studies.

While some studies included in our meta‐analysis appeared to apply a less qualitative methodology, the sensitivity analysis performed revealed this issue to have little impact on the robustness of our findings.

Our research strategy should also be discussed. By carefully selecting study designs, we aimed to capture the specific contribution of serotonergic pathways presumed to underlie OCD. For this purpose, we chose not to include studies providing “nonspecific imagery data” (i.e. imaging techniques that provide brain structural or functional data reflecting undifferentiated neuronal networks). We also excluded studies that investigated brain changes before and after antidepressant treatment since they could potentially modify the effect of OCD on 5‐HT pathways. Then, we chose not to pool some brain regions in the same subgroup to ensure more homogeneous results. Although we chose to group the thalamic and hypothalamic subregions, a sensitivity analysis focusing solely on thalamic data yielded similar results in terms of both effect size and direction. In the same vein, Kambeitz and Howes pooled brainstem, midbrain, pons, substantia nigra, and dorsal raphe in the same group (brainstem group), assuming that these regions could be brought closer structurally or taking into account the issue of low spatial resolution, even though such subregions could encompass distinct physiological roles.^23^ Moreover, the original authors' definitions of regions were not always precisely detailed, and there could have been considerable overlap between regions with different names in different papers, so that grouping by names does not guarantee grouping by anatomically distinct structures.

Another limitation of our work is the presence of several patients with substantial levels of depressive symptoms according to Beck Depression Inventory (BDI) scores in three meta‐analyzed studies (six patients showed BDI scores >16 in Hesse et al.). Given the high overlap between OCD and depression,^64^ this could have affected the results, since Pogarell et al.^31^ found a significant interaction between midbrain SERT binding potential and BDI scores.^31^ Because of the limited number of studies in our meta‐analysis, we could not perform subgroup or meta‐regression analysis to assess the impact of concurrent depressive symptoms on SERT and 5‐HT2A binding among patients with OCD.

Finally, it is important to consider that while molecular imaging results in untreated individuals strongly support the involvement of serotonergic systems in OCD pathophysiology, the observed phenotype may represent a compensatory, rather than primary, process—likely driven by underlying dysregulations in dopaminergic or glutamatergic systems.

Our findings on regional SERT binding alterations suggest potential therapeutic implications, since there are emerging strategies to combine drug delivery with ultrasound‐induced uncaging.^65^ Such an approach could help target drugs that influence serotonergic mechanisms of interest to particular brain regions such as those identified in this work. This could overcome the current limitation of pharmacotherapy strategies that influence the central nervous system as a whole and therefore preclude regional specificity of action.

Finally, by combining results from two decades of molecular imaging studies, we showed that there is robust evidence for a diminution of SERT availability in the midbrain, the brainstem, and the thalamus/hypothalamus regions of the brain of patients with untreated OCD. However, the exact mechanisms underlying this phenotype remain elusive. To deepen our understanding of the involvement of 5‐HT pathways in OCD, forthcoming studies should go beyond the traditional investigation of brain ROIs. It has been proposed that such approaches inadequately capture the intricate anatomy of the 5‐HT system, which includes diverse substructures, making it challenging to clearly identify pathways.^66^

## Disclosure statement

J.L.C. has received honoraria from Janssen, he has participated in advisory boards with Janssen and own stock options from Compass Pathways. I.C. has received a grant from the French Ministry of Health and received honoraria from Lundbeck, MSD, and Lilly. L.M. has received honoraria from Lundbeck and a grant from the French Institute of Research in Public Health. All other authors declare there are no competing financial interests in relation to the work described.

## Author contributions

M.P. and J.L.C. conceived and designed the study, reviewed the articles and drafted the manuscript. B.O. and P.F.P. conducted the statistical analysis. V.B. and L.C. were responsible for methodological aspects concerning nuclear imaging techniques. All authors revised and approved the final version of the article.

## Supporting information


**Data S1.** Supporting information.
