# Supplementary material for: Serotonergic underpinnings of obsessive‐compulsive disorder: A systematic review and meta‐analysis of neuroimaging findings
Source: Psychiatry Clin Neurosci. 2024 Nov 7;79(2):48–59. doi: 10.1111/pcn.13760 (PMC11789457; doi:10.1111/pcn.13760)
Supplement: Supplementary file 1 — Data S1. Supporting information. [file PCN-79-48-s001.docx]

# SUPPLEMENTARY MATERIAL

**SUPPLEMENTARY METHODS:**

**Data sources and search strategy**

With searched in databases using the following equation terms: ‘(OCD OR "obsessive-compulsive disorder"[MeSH Terms]) AND (antidepressant OR SSRI OR clomipramine OR 5-HT1A OR 5-HTT OR SERT OR 5-HT2A OR 5-HT3 OR 5-HT1B OR 5-HT1D OR 5-HIAA OR 5-hydroxytryptamine OR

seroton*) AND (neuroimag* OR imagery OR MRI OR fMRI OR volum* OR structural OR morphometry OR ‘functional magnetic resonance’ OR ‘magnetic resonance’ OR voxel OR imaging OR resting state OR cortical thickness OR grey matter volume OR voxel-based morphometry OR VBM OR diffusion OR DTI OR PET OR positron emission tomography OR SPECT OR positron OR ‘cerebral blood flow’ OR ‘single photon emission computed tomography’ OR ‘arterial spin labeling’ OR ‘voxel- vise’)’.

**Data extraction and quality assessment**

Different outcomes reflecting the same physical variable are often used in studies investigating radioligand binding in the brain. They all rely on a three-compartment model (63) comprising the arterial plasma compartment (Ca), the non-displaceable compartment (C2), and the specific compartment (C3). In this model, the equilibrium distribution volume of a compartment *i* (Vi) is defined as the equilibrium ratio of the tracer concentration in that compartment to the free arterial concentration: *V*i=*C*i/(f1Ca), where f1 equals the free fraction of parent tracer in plasma. V3 is equal to the binding potential (BP) which is the product of the receptor density (Bmax) and affinity (1/KD) (KD being the radioligand equilibrium dissociation constant) (64). V2 represents the non-displaceable uptake, which includes both nonspecifically bound and free ligand. V3″, also known as the specific-to-non-displaceable equilibrium partition coefficient, is defined as V3/V2=BP/V2= Bmax/(KdV2). It is important to note that V3″ is directly proportional to Bmax, provided that both Kd and V2 remain relatively constant across different study populations.

Under equilibrium conditions, V3″ can be calculated as C3/C2. Assuming a referential region (RR) that is mostly devoid of receptors, this parameter can be computed without the need to measure plasma tracer concentrations. It is derived from a ratio of regional count densities: [region-RR]/RR = region/RR – 1 (65). The binding potential quantifies the equilibrium concentration of specific binding as a ratio to another reference concentration (66). BPND refers to the ratio at equilibrium of the specifically bound radioligand to that of non-displaceable radioligand in tissue. BPND compares the concentration of radioligand in a receptor-rich region to a receptor-free region (frequently cerebellum

or occipital region) and is closed to the V3″ previously described. Some authors use equally the term BP2.

Of note, BPND evaluation does not require blood sampling and is relatively easy to implement. BPND could equally be calculated from volume distribution measured with arterial plasma concentration of radioligand. The volume of distribution is the theoretical volume that would be necessary to contain the total amount of an administered drug at the same concentration that is observed in the blood plasma. In radioligand imaging this concept was adapted: the target region was regarded as a particular organ (e.g., the brain) rather than the entire body, and instead of referring to the amount of drug in the entire organ, the target was expressed as the amount of radioligand in a volume of tissue (i.e., a concentration). The volume of distribution is the ratio of the concentration of radioligand in a region of tissue to that in plasma: BPND = (VT-VND)/VND =(VT/VND)-1, where VT = distribution volume of total ligand uptake in tissue relative to the total concentration of ligand in plasma (66). VND is the distribution volume of non- displaceable compartments relative to the total concentration of ligands in plasma. VT/VND can also be defined as the Distribution Volume Ratio (DVR).

In 2007, Innis et al. (68) published a widely-applicable nomenclature paper that simplified previous notations. According to Innis et al., two main outcomes emerge in radioligand imagery: binding potential referred to plasma concentration or BPP, variously called BP, BP1 or V3 in older literature and binding potential referred to the nondisplaceable compartment in brain or BPND, variously referred to as V3" or BP2. The distribution volume of the nondisplaceable compartment, formerly referred to as V2, is VND. Free fraction in plasma, formerly referred to as f1 is fP. The free fraction of the nondisplaceable

compartment, formerly f2, is fND.

In the interest of clarity, we chose to use the modern nomenclature throughout the text and in Table 1. For this purpose, many of the outcomes used in the older literature prior to the adaptation of uniform nomenclature were named as either equivalent to BPND or BPP . For example, papers using DVR (Hesse et al., 2011 and Müller-Vahl et al., 2019) were presented as using BPND (taking into account that BPND = DVR – 1), as well as papers using V3” (Pogarell et al., 2003; Stengler-Wenzke et al., 2004; Van der Wee et al., 2004; and Hesse et al., 2005) and BP2 (Hasselbach et al., 2007). This was also the case for Perani et al. (2008) and Wong et al. (2008), who used an unspecified ‘BP’ outcome corresponding to BPND according to the modern nomenclature because of the use of a cerebellar reference region and the absence of arterial input functions. Conversely, the binding outcome in Simpson et al. (2003) was identified as equivalent to BPP, as well as the DV’3 used in Adams et al. (2005), because DV’3 = (CROI − Ccerebellum)/Cplasma.

**Meta-analysis**

We conducted separate meta-analyses for different brain regions. For this purpose, we designed groups of ROIs a priori, considering their close anatomical and/or physiological relationships. For example, we grouped ROIs such as 'thalamus' and 'thalamus/hypothalamus' together, since molecular imaging studies often provide binding values for a region encompassing these two anatomically adjacent subregions, and no study focused on hypothalamus solely (see Discussion). When ‘thalamus’ and ‘hypothalamus’ ROI were investigated separately in the same study (25), we chose to include only the ‘thalamus’ ROI in our meta-analysis, since we consider this subregion as the most meaningful

considering OCD pathophysiology. To ensure the robustness of our results, we conducted a sensitivity

analysis including only studies focusing on thalamus subregion solely.

Similarly, we combined 'cingulate' and 'anterior cingulate' ROIs, 'ventral striatum' and 'nucleus accumbens' ROIs, and 'parietal cortex' and 'inferior parietal lobule' ROIs in the same subgroup, considering their shared anatomical and functional characteristics. Even though there might have been some differences in the way these ROIs were delineated across studies, we considered the shared denomination for grouping. This grouping method has been previously used in the field (23).

To minimize methodological biases, we decided not to regroup ROIs in the principal analyses when we were uncertain whether they covered the same brain region. For instance, we refrained from combining ROIs like 'raphe nucleus', 'pons', 'midbrain-pons', and 'brainstem'; or 'striatum' and 'ventral striatum' because we were not entirely confident about their exact delineation across different studies. This cautious approach aimed to maintain the integrity of the data and avoid potential inaccuracies in the analyses. Of note, we followed a method previously used to calculate the average binding values of substructures separated in different ROIs when possible (17). For instance, we used the commune variance method to merge ‘left thalamus’ and ‘right thalamus’ ROIs from the Van der Wee et al. study

(28). We proceeded this way since the inter-ROIs variance was low so the total variance can be approximated by the inter-individual variance.

# SUPPLEMENTARY RESULTS:

**Description of studies:**

## Serotonin transporter (SERT) studies

Pogarell et al. (2003) utilized SPECT imaging to measure [123I]-beta-CIT binding potential in the midbrain-pons regions of OCD patients (41). The results showed higher binding potential values in the patient group compared to healthy controls (HC) (2.26±0.37 for HC vs. 1.81±0.23 for OCD, p=0.003). This difference remained statistically significant even after controlling for age, gender, and Y-BOCS scores. While there was no significant association between binding values and Y-BOCS scores, there was a significant inverse association between beta-CIT binding and depression scores on the Beck Depression Inventory (BDI) (r=-0.69, p=0.04). Upon further investigation through subgroup analysis, the difference in SERT binding potential between patients and controls was only significant for the early-onset subgroup, where OCD was present before 18 years of age.

Stengler-Wenzke et al. (2004) conducted a comparison of [123I]-beta-CIT binding potential between individuals with OCD and HC, focusing on thalamic/hypothalamic, midbrain, and brainstem regions (42). The study revealed lower binding potential values in the midbrain (3.51 ± 0.45 vs 4.89 ± 1.23; p<0.005) and the brainstem (2.38 ± 0.76 vs 3.53 ± 1.01; p<0.05) for the OCD group. However, no association was observed between binding values and Y-BOCS scores.

In another SPECT study using [123I]-beta-CIT radiotracer, Van der Wee et al. did not find a significant difference in binding potential values between individuals with OCD and HC across three brain regions (left and right thalamus, midbrain, and pons) (28). Moreover, there was no association between binding values and Y-BOCS scores in the patient group.

Hesse et al. (2005) reported a reduction in [123I]-beta-CIT binding potential in the thalamus/hypothalamus (p=0.026), midbrain (p=0.008), and brainstem regions (p=0.014) of individuals with OCD (43). Notably, a negative association was found between [123I]-beta-CIT binding potential in the hypothalamus/thalamus regions and Y-BOCS score (r=-0.79, p=0.001 after correcting for age and duration of illness).

In contrast, Hasselbalch et al. observed a reduction in [123I]-beta-CIT binding potential in the midbrain-pons region of OCD patients compared to HC (0.97±0.07 vs 0.84±0.12; p=0.011) (44). These results remained consistent even after adjusting for age and gender. However, no association was found between binding values and the symptomatic severity of OCD.

Zitterl et al. (2007) investigated [123I]-beta-CIT binding potential using SPECT imaging in the thalamic and hypothalamic brain regions of both OCD patients and HC (45) The patient group was homogeneous in terms of clinical subtypes, as all participants presented with checking compulsions. The patients had significantly lower binding potential values compared to HC (1.38±0.19 vs 1.69±0.21; p<0.001). Moreover, the authors identified a significant negative correlation between [123I]-beta-CIT binding in the thalamus/hypothalamus region and the Y-BOCS scores of the patients (r=-0.80, p<0.001). In a multivariate model, the authors found that the severity and the duration of illness had a considerable impact on [123I]-beta-CIT binding values (B=-0.03±0.005, p<0.001 and B=0.006±0.002, p<0.05, respectively).

In the study by Matsumoto et al., [^11^C]DASB PET imaging was used to investigate SERT binding potential across multiple brain regions in OCD patients and HC (46). Among the different ROIs, there were statistical differences between groups concerning the orbitofrontal cortex (0.13±0.05 for HC vs 0.08±0.02 for OCD; p=0.0005), the temporal cortex (0.43±0.12 vs 0.34±0.03; p=0.007) and the insular cortex (0.43±0.11 vs 0.30±0.04; p=0.0008). There was no significant association between binding values and Y-BOCS scores in any region.

In a 2007 study using the [^11^C]DASB radiotracer, Reimold et al. retrieved a significant reduction of SERT binding potential in the midbrain (2.44±0.33 vs 2.87±0.58) and the thalamus (1.14±0.24 vs 1.35±0.18) of OCD patients as compared to HC (47). Adjusting for diagnosis, age, gender and smoking status in a multiple regression analysis did not change the results. There was an association between SERT binding values and Y-BOCS scores (thalamus binding and age explained 83% of Y-BOCS variance). Notably, patients in this study had mean BDI scores of 15.1±11.9.

Hesse et al. (2011) conducted a study comparing SERT binding potential values across multiple brain regions using [11C]DASB SPECT imaging in two subgroups of OCD patients: the early-onset OCD (EO-OCD), diagnosed at 17 years old or before, and the late-onset OCD (LO-OCD), alongside HC (25). Among the OCD patients, 5 individuals had BDI scores ≥17. The results revealed significantly lower SERT binding potential in the LO-OCD group compared to HC in several brain regions, including the raphe, midbrain, hypothalamus, thalamus, striatum, hippocampus, amygdala, medial prefrontal cortex, and occipital cortex (all p<0.05 for t-tests). Additionally, lower binding potential were observed in LO-OCD compared to EO-OCD in some regions, specifically the midbrain, thalamus, striatum, hippocampus, and occipital cortex (all p<0.05 for t-tests). However, there were no significant differences in binding potential values between EO-OCD and HC. Univariate ANCOVA analysis indicated a difference in binding between the entire OCD patient group and HC specifically in the anterior cingulate cortex region (p=0.02), and differences between EO-OCD and LO-OCD were observed in the nucleus accumbens (p=0.04), amygdala (p=0.02), and putamen regions (p=0.01). However, multivariate ANCOVA analysis showed no significant differences between the overall OCD group and HC. Furthermore, there was no association observed between binding potential and Y-BOCS scores in any brain region for the OCD group as a whole, as well as within the EO-OCD and LO-OCD subgroups.

Simpson et al. used [^11^C](+)McN-5652 radiotracer to evaluate SERT binding potential in the midbrain, the thalamus, the hippocampus, the amygdala, the anterior cingulate cortex, and across striatal subregions (24). There were no statistically significant differences in SERT binding potential between OCD and HC in any of the above-mentioned regions, nor associations between these values and OCD severity.

Wong et al. investigated both SERT and 5-HT2AR binding potentials in a sample of patients with OCD and Tourette Syndrome (TS) using [11C](+)McN-5652 and [11C]MDL100,907 radiotracers, respectively (37). There was a significant decrease of SERT binding potential in the midbrain (p<0.05) of patients diagnosed with OCD and a comorbid TS disorder (TS+OCD group), as compared to HC. The authors didn’t provide statistical comparisons between binding potential values in the 9 patients presenting both OCD and TS and 2 patients presenting TS only.

In a similar work, Müller-Vahl et al. investigated SERT binding potential via [^123^I]ADAM SPECT in 8 TS+OCD, 10 TS-OCD, 5 OCD, and 10 HC (36). To note, 3 TS+OCD patients and one pure OCD patient were diagnosed with MDD. The SERT binding potential at baseline was increased in the caudate nucleus (p=0.0284), hypothalamus (p=0.0227), and midbrain (p=0.0191) of TS+OCD patients as compared to the HC group. When they compared the values for the TS+OCD patients to those of TS- OCD patients, there was an increase in SERT binding potential in the caudate nucleus (p=0.0216), the midbrain (p=0.0479) and the thalamus (p=0.0491). No variation in SERT binding was found within the OCD-only group compared to other groups. Additionally, no correlation was observed between SERT binding and Y-BOCS ratings.

## Serotonin receptors (5-HTR) studies

Adams et al. used the radiotracer [^18^F]-altanserin (a 5-HT2AR antagonist) to study cerebral 5- HT2AR binding potential in 15 patients with OCD (39). Compared to 15 HC, there was a higher binding potential in the caudate nuclei of OCD patients (0.24 ± 0.14 vs 0.15 ± 0.13, p<0.05). After treatment with an SSRI for at least 12 weeks, the difference disappeared. No association was found between the severity of OCD symptoms (Y-BOCS score) and the DV for [^18^F]-altanserin binding.

Perani et al. used the [11C]MDL100,907 radiotracer (a 5-HT2AR antagonist) to investigate brain regions in a cohort of 9 drug-naïve OCD patients and 15 matched HC (48). In the voxel-based analysis, there were multiple areas across cortical regions (frontal and cingulate cortices) showing statistically significant reduced [11C]-MDL binding potential in OCD patients compared to HC. The authors found an inverse association between Y-BOCS scores and binding potential in the frontal cortex (orbitofrontal and dorsolateral), temporal cortex (lateral and medial), and the inferior parietal lobule of OCD patients (simple regression model using SPM2 software).

Simpson et al. conducted the same design as mentioned above, consisting of [11C]MDL100,907 binding potential measurements across cortical and limbic regions (49). There were neither differences in 5-HT2AR availability approached by binding potential scores in OCD versus HC, nor an association

between OCD severity (Y-BOCS) and binding potential in any region (ROI and voxel-wise). The only statistically significant finding is an inverse association between the binding potential in OFC and the age at onset in OCD patients, after adjusting for age and multiple comparisons (Pearsons’s ρ = −0.68, p

= 0.002).

Wong et al. investigated 5-HT2AR binding potential in a sample of patients with OCD and TS using [^11^C]MDL100,907 (37). There was no significant difference between groups (TS+OCD vs TS- OCD vs HC) in regard to 5-HT2AR binding potential.

Finally, Pittenger et al. conducted a unique study utilizing a 5-HT1BR ligand ([^11^C]p943) to assess the receptor's availability in 12 OCD patients and HC (50). Additionally, they measured pre-pulse inhibition (PPI) in 10 patients and controls. Although there were no significant differences in 5-HT1BR binding potential between patients and controls or associations with Y-BOCS scores after controlling for the false discovery rate, the authors emphasized that the relationship (association) between regional binding potential and PPI scores differed in the OCD and control groups. Specifically, the results from a mixed model analysis showed a statistically significant interaction between group (OCD vs HC), region (cortical vs subcortical ROI), and PPI scores on [11C]-P943 binding potential values (F(1,16)= 10.30, p=0.0055).

## Serotonin synthesis studies

Berney et al. conducted a study investigating 5-HT synthesis using alpha[11C]methyl-L- tryptophan (α-[^11^C]MTrp) trapping constant (K, in ml/g/min) measurements via PET imaging in a sample of 21 OCD patients (51). The researchers discovered a statistically significant increase in voxel- wise K in OCD patients compared to HC, particularly in the right hippocampus and left inferior temporal gyrus regions. This finding was further confirmed by ROI-based analysis, where the results of the group x hemisphere analysis of variance for K showed significant differences in the right hippocampus (F(1,40)= 14.75, p<0.001) and inferior temporal gyrus (F(1,40)= 7.97, p<0.007). A subgroup analysis, limited to male participants, revealed a noteworthy increase in K values in the caudate nuclei of OCD patients compared to HC (F(2,39)= 9.06, p<0.01). Furthermore, the authors identified a positive and

statistically significant association between the radiotracer trapping values in the temporal gyri and the Y-BOCS scores in OCD patients, using SPM8 software. A similar association was observed between clinical severity and right caudate trapping values in male patients.

## Other relevant designs

Kim et al. conducted a fMRI resting state study on 102 OCD patients and matched controls to investigate the functional connectivity (FC) of the raphe nuclei (RN) (38). 6 patients presented MDD. Analysis showed a larger FC between the RN and various cortical and subcortical ROI (temporal gyri, paracingulate gyrus, amygdala, hippocampus, putamen, caudate, thalamus, and brainstem) in OCD as compared to HC. Only one cluster (the left occipital pole) showed lower FC in OCD vs HC (p=0.01). A trending mild positive association existed between Y-BOCS and FC linking RN and left infero-medial temporal gyri (r=0.197, p=0,05 after controlling for baseline Hamilton Scale for Depression and Hamilton Scale for Anxiety scores). Regression analysis based on 54 patients of the sample that were treated by SSRI only and followed for 16 weeks revealed that a smaller FC linking RN and medio- superior temporal gyri was a predictor of clinical improvement (the *R²* for association between % of clinical improvement measured by Y-BOCS and FC is equal to 0.317, p=0.004). Furthermore, the authors found a greater FC linking RN and medio-superior temporal gyri in non-responders as compared to responders (p<0.05).


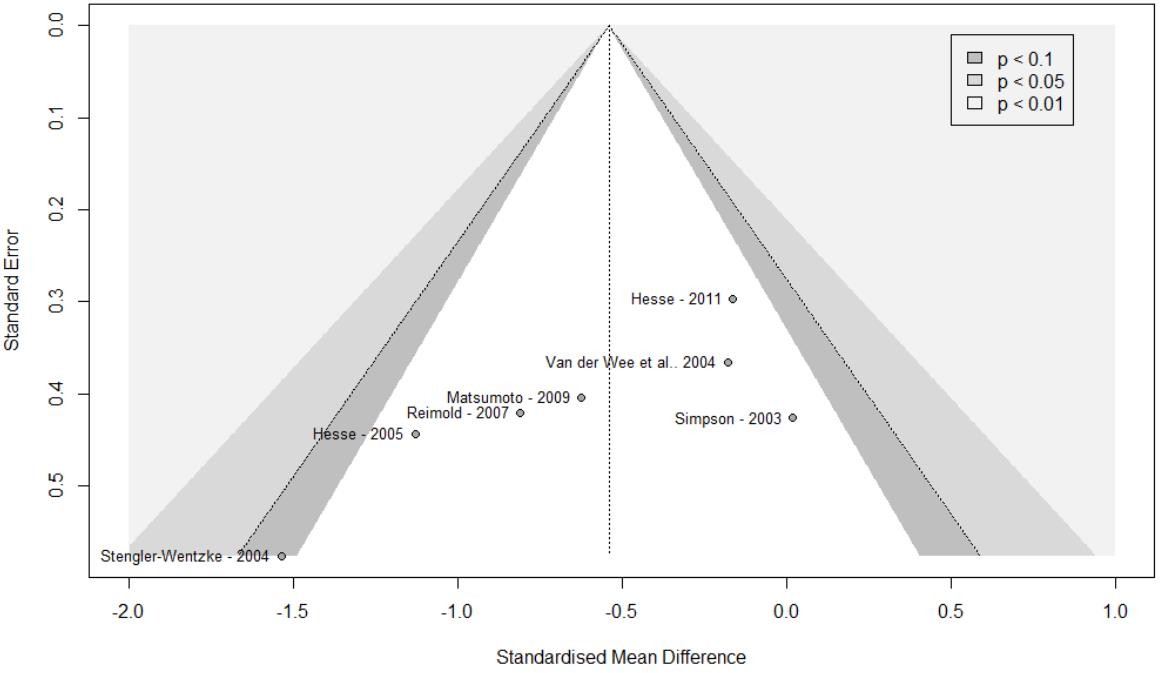


**Figure S1: funnel plot (midbrain ROI, SERT binding)**


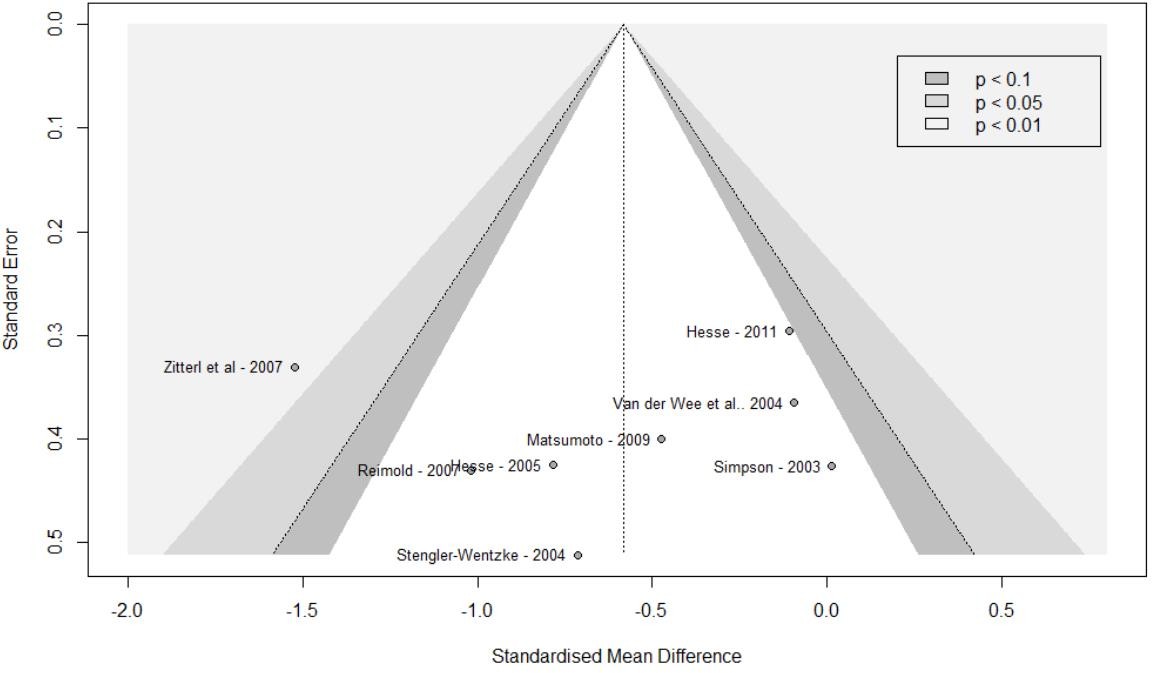


**Figure S2: funnel plot (thalamus ROI, SERT binding)**


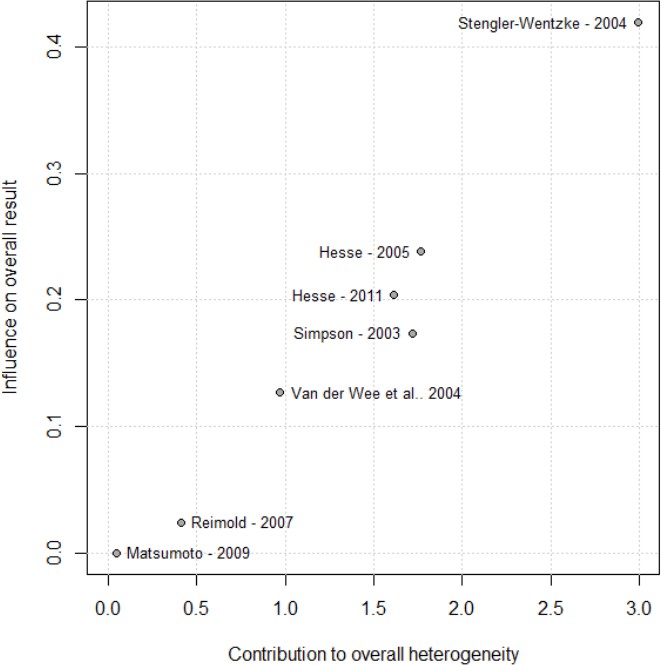


**Figure S3: Baujat plot (midbrain ROI, SERT)**

The study by Stengler-Wenzke et al. (2004) has the most significant impact on heterogeneity, and is the most influential study on the overall results. There is no study in the bottom right corner that should be excluded from the analysis.


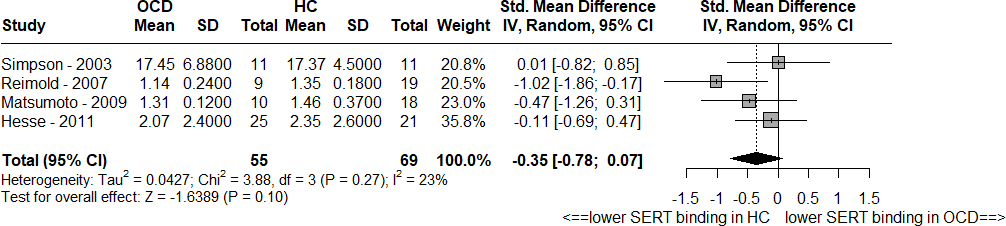


**Figure S4 – sensitivity analysis : SERT binding in thalamus ROI only**


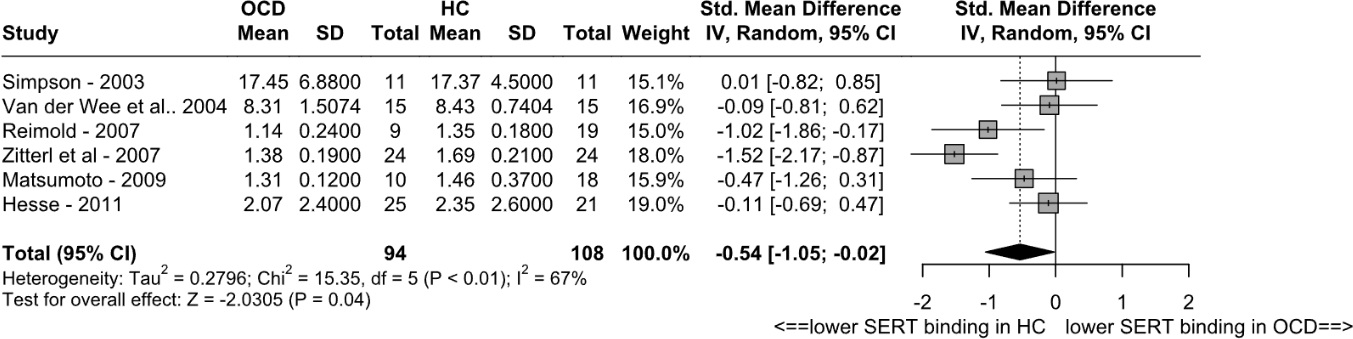


**Figure S5 – sensitivity analysis : exclusion of low quality studies for SERT binding in thalamus/hypothalamus ROI only**
